# The natural history of EGFR and EGFRvIII in glioblastoma patients

**DOI:** 10.1186/1479-5876-3-38

**Published:** 2005-10-19

**Authors:** Amy B Heimberger, Dima Suki, David Yang, Weiming Shi, Kenneth Aldape

**Affiliations:** 1 Department of Neurosurgery, The Brain Tumor Center, The University of Texas M. D. Anderson Cancer Center, Houston, Texas, USA; 2 Pathology, The Brain Tumor Center, The University of Texas M. D. Anderson Cancer Center, Houston, Texas, USA

**Keywords:** Glioma, survival, sub-total resection

## Abstract

**Background:**

The epidermal growth factor receptor (EGFR) is over expressed in approximately 50–60% of glioblastoma (GBM) tumors, and the most common EGFR mutant, EGFRvIII, is expressed in 24–67% of cases. This study was designed to address whether over expressed EGFR or EGFRvIII is an actual independent prognostic indicator of overall survival in a uniform body of patients in whom gross total surgical resection (GTR; ≥ 95% resection) was not attempted or achieved.

**Methods:**

Biopsed or partially/subtotally resected GBM patients (N = 54) underwent adjuvant conformal radiation and chemotherapy. Their EGFR and EGFRvIII status was determined by immunohistochemistry and Kaplan-Meier estimates of overall survival were obtained.

**Results:**

In our study of GBM patients with less than GTR, 42.6% (n = 23) failed to express EGFR, 25.9% (n = 14) had over expression of the wild-type EGFR only and 31.5 % (n = 17) expressed the EGFRvIII. Patients within groups expressing the EGFR, EGFRvIII, or lacking EGFR expression did not differ in age, Karnofsky Performance Scale (KPS) score, extent of tumor resection. They all had received postoperative radiation and chemotherapy. The median overall survival times for patients with tumors having no EGFR expression, over expressed EGFR only, or EGFRvIII were 12.3 (95% CI, 8.04–16.56), 11.03 (95% CI, 10.18–11.89) and 14.07 (95% CI, 7.39–20.74) months, respectively, log rank test p > 0.05). Patients with tumors that over expressed the EGFR and EGFRvIII were more likely to present with ependymal spread, 21.4% and 35.3% respectively, compared to those patients whose GBM failed to express either marker, 13.0%, although the difference was not statistically significant. There was no significant difference in multifocal disease or gliomatosis cerebri among EGFR expression groups.

**Conclusion:**

The over expressed wild-type EGFR and EGFRvIII are not independent predictors of median overall survival in the cohort of patients who did not undergo extensive tumor resection.

## Background

Glioblastoma multiforme (GBM) is the most common primary malignant neoplasm of the central nervous system in adults. Despite multimodal therapies, the median survival time of patients with GBM is approximately 1 year; however, there is considerable variability among these patients. Prognostic indicators have included age [1,2], Karnofsky Performance Scale (KPS) score [3], and extent of surgical resection [4,5]. The most frequent genetic alteration associated with GBM is amplification of the epidermal growth factor receptor (EGFR) gene, which results in over expression of the EGFR, a transmembrane tyrosine kinase receptor [6]. The majority of GBMs with EGFR amplification also contain the mutant EGFR gene, EGFRvIII [7], which is characterized by the deletion of exons 2–7, resulting in an in frame deletion variant that has a truncated extracellular domain with ligand-independent constitutive activity [8]. Previous work has shown that EGFR amplification is evident in all GBMs expressing EGFRvIII and GBMs lacking the amplified EGFR are not positive for EGFRvIII protein. In addition, we have previously shown that the positive staining with the 528 antibody, which recognizes an unspecified extracellular epitope of the EGFR, is highly correlated with EGFR amplification [9].

The role of the over expressed EGFR (wild type) and the variant (vIII) receptor in malignant progression of glial tumors and their respective impacts on overall survival have been debated in the literature. Over expression of wild-type EGFR was not found to be an independent prognostic indicator of survival in several studies [10-12], and one study was inconclusive [13]. Four studies identified EGFR as a negative prognostic indicator of survival [14-16], one of which showed the effect only in patients younger than 45 [17]. In some of these studies, analysis was limited by small sample size, uncharacterized extent of surgical resection, and variable postoperative treatment. The prognostic impact of EGFRvIII has not been as extensively studied, but in the study that addressed this variable, the presence of EGFRvIII was found to be an independent and significant unfavorable prognosticator of survival [18]. In contrast, our group has shown that the presence of the over expressed EGFR and EGFRvIII were not independent negative prognostic indicators in patients who were able to undergo gross total resection (GTR)(>95% MRI-based volumetric resection) [19] when confounding variables were accounted for. However, our previous study had an inherent bias in that more invasive, infiltrative tumors were less likely to be selected for surgery and less likely to have a GTR. Thus, the primary purpose of this study was to determine if EGFR and EGFRvIII are negative prognostic indicators in patients who either receive a biopsy, partial resection (<85%) or subtotal resection (85–95%).

EGFR amplification and EGFRvIII have been shown to increase glioma proliferation and invasion *in vitro *[8,9]; therefore logically EGFR and/or EGFRvIII expression could exhibit a proclivity towards the development of multifocal disease, gliomatosis cerebri or ependymal seeding. Therefore, the secondary purpose of this study was to address the natural history of EGFR and EGFRvIII expression in GBM patients undergoing less than GTR.

## Methods

### Study Population

The study was conducted according to an IRB-approved protocol (LAB03-0228). 54 GBM (WHO grade IV) patients received conformal irradiation and adjuvant chemotherapy and were retrospectively reviewed to determine whether tumor expression of EGFR or EGFRvIII conferred a poor prognosis. Clinical and survival information was obtained from the Department of Neurosurgery Clinical and Imaging Database and The University of Texas M. D. Anderson Cancer Center Tumor Registry. All tissue specimens were acquired at initial diagnosis and resection and were classified morphologically and graded according to WHO criteria.

### Immunohistochemical Detection of Over Expressed EGFR and EGFRvIII

Immunostaining was performed as previously described [17]. Briefly, 5-μm tumor-tissue sections were mounted on positively charged slides, deparaffinized, and rehydrated in phosphate-buffered saline (PBS). Endogenous peroxidase activity was blocked with 3% hydrogen peroxide in PBS/0.05% Tween 20 for 20 min. Sections were washed in PBS and blocked for 20 min in the appropriate serum (from the same species as the secondary antibody) diluted to 10% in PBS. The primary antibody for EGFR detection was the monoclonal mouse anti-human pan-EGFR clone 528 (Oncogene Research; 1:50 dilution) [20] and for EGFRvIII detection was a rabbit anti-human polyclonal antibody (Zymed, San Francisco, CA; 1:1200 dilution). For EGFRvIII staining, microwave antigen retrieval was performed by placing the slides in 50 mM citrate buffer (pH 6.0) and microwaving for 12 min at full power and 10 min at 20% power, followed by cooling for 15 min and two to three 5-min washes in PBS. For EGFR staining, pretreatment consisted of placing 0.025% trypsin on the tissue and incubating for 30 min at room temperature. Primary antibodies, diluted in PBS/10% serum, were applied to the sections in a humid chamber overnight at 4°C. Sections were washed two to three times in PBS, and secondary antibodies were applied using the Dako (Carpinteria, CA) Envision kit, according to the manufacturer's instructions. Detection of bound secondary antibody was performed with diaminobenzadine for 5 min. Sections were then counterstained with hematoxylin and mounted. Each batch of stained slides was accompanied by a positive control (as determined by both positive staining for EGFR and EGFRvIII), as well as demonstration of gene amplification using samples previously described as positive by both genetic analyses (Southern analysis and RT-PCR) as well as immunohistochemistry [9]. Non-neoplastic brain tissue was used as a negative control, and positive staining was never seen in this tissue. As additional controls, 20 randomly selected cases were stained using equal concentrations of mouse (anti-cytokeratin 14, Biogenex, San Ramon, CA) and rabbit antibodies (anti-cytokeratin 19, Neomarkers of Lab Vision Corporation, Fremont, CA) at concentrations matching the EGFR and EGFRvIII staining conditions, respectively. These irrelevant controls were uniformly negative in each of the 20 cases tested. Scoring was accomplished using a simple positive-negative scoring system. Any detectable cytoplasmic-membrane staining in tumor cells was scored as positive/overexpressed.

### Statistical Analysis

The frequencies and descriptive statistics of demographic and clinical variables were performed for the patients in this study. The chi-square or exact test (StatXact 3 for Windows) was used for categorical variables as appropriate. The analysis of variance was used for continuous variables. Cumulative survival times from the time of surgery at our institution were computed using the Kaplan-Meier method [21]. Overall survival curves for the various subgroups were compared using the log rank test. The Cox proportional hazards model was used to obtain crude rate ratios, adjusted rate ratios and their 95% confidence intervals for the various EGFR categories [22]. Adjustments were done for age, sex, KPS score, radiographic enhancement, radiographic necrosis, extent of edema, midline shift, multifocal disease or gliomatosis cerebri and ependymal involvement.

## Results

### Demographic Characteristics

In our study of 54 GBM patients, 43% (n = 23) failed to express the EGFR, 57% (n = 31) were positive for the pan-EGFR stain and of those that expressed EGFR, 31% (n = 17) also expressed the EGFRvIII variant while 26% (n = 14) failed to express EGFRvIII. Staining for EGFR was typically diffuse, while the staining for EGFR vIII was generally more focal (not shown) as has been previously reported [23]. This distribution of expression was similar to those GBM patients who underwent GTR [19]. There was no significant difference in age, KPS score, or extent of surgical resection (biopsy, subtotal or partial resection) among the patients whose tumors failed to express EGFR, over expressed the wild-type EGFR, or expressed EGFRvIII (Table 1). Interestingly, men were more likely to over express EGFR only (79%) or EGFRvIII (88%). This was not a trend observed in GBM patients who had undergone GTRs [19].

**Table 1:** Demographic characteristics of patients with glioblastoma multiforme categorized according to epidermal growth factor receptor expression of the tumor

**Parameter**		**EGFR^a ^negative**	**EGFRwt positive only**	**EGFRvIII positive**
Total 54, N (%)		23 (43)	14 (26)	17 (31)
*Sex, N (%)	M	15 (65)	11 (79)	15 (88)
	F	8 (35)	3 (21)	2 (22)
*Age, years, Median (range)		57 (15–79)	65 (25–71)	59 (54–73)
*KPS score, Median (range)		90 (50–100)	80 (50–90)	90 (60–100)

^a^EGFR, epidermal growth factor receptor; wt, wild type; vIII, vIII mutant; KPS, Karnofsky Performance Scale.

While there was a trend towards increased post-surgical incidence of pulmonary embolus in patients who expressed the EGFRvIII (17.6%) compared to those who over expressed the EGFR only (7.1%) or failed to express EGFR (8.7%), it was not statistically significant (p = 0.36). No such trend was observed with DVT.

### Radiographic Characteristics

There was no significant difference in the location, extent of necrosis, amount of MR image contrast-enhancement, extent of edema, or amount of brain midline shift among the three EGFR expression categories of GBMs (Table 2). There was no significant difference in the ratio of MRI T2-bright volume to T1-enhancing volume between EGFR expression groups.

**Table 2:** Radiographic characteristics and post surgical events in glioblastomas according to epidermal growth factor receptor expression category

**Parameter**		**EGFR^a ^negative**	**EGFRwt positive**	**EGFRvIII positive**
Total of 54, N (%)		23 (43)	14 (26)	17 (31)
Cortical matter involvement, N (%)				
Yes		17 (74)	10 (71)	11 (65)
No		6 (26)	4 (29)	6 (35)
Ependymal, N (%)	yes	3 (13)	3 (21)	6 (35)
Multifocality, N (%)	Yes	2 (9)	2 (14)	2 (12)
Gliomatosis cerebri, N (%)	Yes	5 (22)	4 (29)	3 (18)
*Severe necrosis (grade 3), N (%)	Yes	5 (24)	6 (46)	7 (44)

^a^EGFR, epidermal growth factor receptor; wt, wild type; vIII, vIII mutant; *radiographic imaging not available for all patients.

### Natural Disease History Based on EGFR and EGFRvIII Expression in GBMs

Patients that over expressed the EGFR or expressed the EGFRvIII presented with a greater incidence of ependymal spread, 21.4% and 35.3% respectively, when compared to tumors that failed to express either marker 13.0% but this was not statistically significant. Ependymal spread negatively impacted median survival in EGFR expressing (5.3 versus 11. months) and EGFRvIII expressing (8.3 versus 15 months) GBM patients. There were no significant differences in the percentages of patients who had multifocal disease or gliomatosis cerebri, irrespective of EGFR or EGFRvIII expression status. However, there was an 18–22% incidence of gliomatosis cerebri in all EGFR expression categories in the less than GTR patient group compared to only 3–5% incidence in those patient who underwent GTR.

### Impact of EGFR or EGFRvIII on Survival

**Figure 1:**
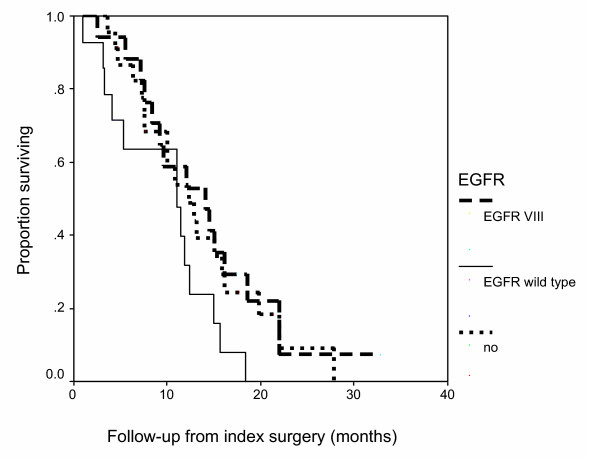
**Graph showing Kaplan-Meier estimates of overall survival in glioblastoma multiforme patients who underwent sub-total resection followed by standard-of-care radiation therapy and chemotherapy**. Patients with tumors not expressing the epidermal growth factor receptor (EGFR; n = 23; solid black line), expressing amplified EGFR (n = 14; dashed grey line), and expressing EGFRvIII (n = 17; dotted black line) had median overall survival times of 12.3 (95% CI, 8.04–16.56), 11.03 (95% CI, 10.18–11.89), and 14.07 (95% CI, 7.39–20.74) months, respectively, which were not statistically significantly different.

Over expressed wild-type EGFR or EGFRvIII were not independent predictors of overall survival and did not confer a worse prognosis (Figure 1). The median overall survival times for patients with tumors having no EGFR expression, over expressed EGFR only, or EGFRvIII were 12.3 (95% CI, 8.04–16.56), 11.03 (95% CI, 10.18–11.89) and 14.07 (95% CI, 7.39–20.74) months, respectively, indicating that neither EGFR or EGFRvIII were negative prognostic indicators in GBM patients unable to undergo GTR. The median overall survival times for patients who underwent GTR with tumors having no EGFR expression, over expressed EGFR only, or mutant EGFRvIII was 11.68, 11.9 and 13.0 months, respectively indicating that the median survival were not significantly different from those patients who underwent GTR. Our group has previously demonstrated that the extent of surgical resection impacts survival in a series of 416 patients with glioblastoma multiforme [4]. The statistically significant impact on survival started in those patients who received 89% volumetric resections or greater. The vast majority of patients in our current study were subtotal resections defined as extents of resection of 85% to <95%, as opposed to <85%. This could account for the comparable survival to the GTR cohort (≥ 95% resection). There was no significant difference in age or KPS between patients who underwent GTR and those who did not.

### Established Prognostic Factors

Our results were consistent with prior findings demonstrating that age ≥ 65 years has a negative prognostic impact on survival although this difference did not reach statistical significance, p = 0.33). This was likely due to our small sample size and a paucity of young and very old patients (n = 4 under 40 years; n = 2 over 75 years). KPS score was found to be an independent prognostic indicator in our study, consistent with previous studies that have validated KPS as a prognostic indicator in GBM patients. Radiographically visualized necrosis was more common in EGFR-expressing tumors but the sample size was insufficient to ascertain if necrosis was a negative prognosticator.

## Discussion

Despite previous reports indicating that EGFR and EGFRvIII were negative prognostic indicators, we found that neither the over expressed wild-type EGFR nor EGFRvIII were independent predictors of median overall survival in GBM patients who underwent GTR (≥ 95% volumetric resection). The median overall survival times for patients who had tumors devoid of EGFR expression, with over expression of EGFR, or with mutant (EGFRvIII) expression were 0.96, 0.98, and 1.07 years, respectively. In the study by Shinojima et al. [18], the authors concluded that EGFR amplification in GBMs was associated with shorter patient survival in a heterogeneous group of patients who underwent a wide variety of treatments including gross-total resection, partial resection, and biopsy. We therefore hypothesized that the EGFR and EGFRvIII may have a negative prognostic impact in patients that are not able to undergo GTR. This would be one potential variable to account for the discrepancies between the results. However, we did not see a difference in median survival across EGFR expression categories in the subcategory of patients unable to undergo GTR.

As others have found there was a range in the positive cases with respect to the proportion of tumor cells which were positive. This was especially true for EGFRvIII [23]. The number of samples in this study was relatively small given the restriction to subtotally resected cases, and therefore stratification by proportion of positively stained cells was not attempted. However, future studies examining this issue in EGFRvIII-positive tumors are warranted.

We investigated the possibility that the absence of an EGFR prognostic effect could be explained by the age distribution of our sample. Simmons et al. [17] had demonstrated that EGFR over expression was an unfavorable prognostic factor in patients less than 55 years of age. In the study by Shinojima et al. [18], 97% of the patients were <70 years old. If EGFR over expression is truly an unfavorable prognostic factor in younger age patients, such an age distribution may have been sufficient to influence the authors' conclusion that EGFR over expression impacted survival rates independently of age. In our study, however, the respective median overall survival times for our patients under 55 years of age whose tumors expressed neither type of EGFR, overexpressed wild type EGFR or expressed EGFRvIII, were not statistically different. In our earlier study of patients who underwent GTR we saw a trend toward a negative effect of EGFR and EGFRvIII expression on survival in patients under 40 years of age. However, no conclusion could be drawn for this subgroup in this study as there were only 4 patients aged <40. Though age bias is an unlikely explanation for the negative findings in our study, the differential effect of EGFR within different age groups requires further investigation.

EGFR and EGFRvIII expression have been shown to increase the infiltrative and invasive properties of glioma cells [24,25], therefore, one could hypothesize that patients expressing these markers may be more likely to present with multifocal disease, ependymal dissemination, or gliomatosis cerebri and perhaps this is the confounding variable between the studies accounting for the discrepancy in prognostic impact. GTR is not possible when there is multifocal disease, extensive ependymal spread or gliomatosis cerebri. Patients deemed "unresectable" due to extensive tumor invasion and multifocality of disease may have been more likely to have the over expression of the EGFR or the EGFRvIII. Additionally, these characteristics on radiographic presentation typically influence the treatment options toward biopsy and palliative treatment modalities. Although there was a trend of increased ependymal involvement within tumors expressing EGFR (21%) and EGFRvIII (35%) compared with tumors not expressing EGFR (13%) in patients not undergoing GTR, this was not statistically significant. The study sample was too small to determine if there was a statistically significant difference in the incidence of gliomatosis cerebri or multifocal disease. We did observe an increased incidence of gliomatosis cerebri in GBM patients that did not undergo GTR compared to those that were able to undergo GTR. These factors were not addressed in the other studies and could potentially account for the differences in EGFR and EGFRvIII prognostic impact between these studies, especially if there was a significant incidence of these in a small series. Alternatively, there may be unidentified confounding variable that our studies and the other published ones did not account for.

## Conclusion

Within the subcategory of patients with GBM who underwent less than GTR, the presence of EGFR or EGFRvIII as a single mutation does not account for poor prognosis; however, simultaneous molecular genetic changes with prognostic significance have not been addressed in this study.

## Declaration of competing interests

The author(s) declare that they have no competing interests.

## Authors' contributions

ABH conceived the project, designed and coordinated the study and drafted the manuscript. DS designed the database and carried out the data analysis. DY participated in the database design and assisted with the immunohistochemistry. WS performed the detailed volumetric and radiographic analysis. KA performed and interpreted the immunohistochemistry. All authors read and approved the final manuscript.
